# OrganoLabeler: A Quick and Accurate Annotation Tool for Organoid Images

**DOI:** 10.1021/acsomega.4c06450

**Published:** 2024-11-06

**Authors:** Burak Kahveci, Elifsu Polatli, Yalin Bastanlar, Sinan Guven

**Affiliations:** 1 Izmir International Biomedicine and Genome Institute, Dokuz Eylul University, Izmir 35340, Türkiye; 2 Izmir Biomedicine and Genome Center, Izmir 35340, Türkiye; 3 Department of Computer Engineering, Izmir Institute of Technology, Izmir 35430, Türkiye; 4 Faculty of Medicine, Medical Biology and Genetics Department, Dokuz Eylul University, Izmir 35340, Türkiye

## Abstract

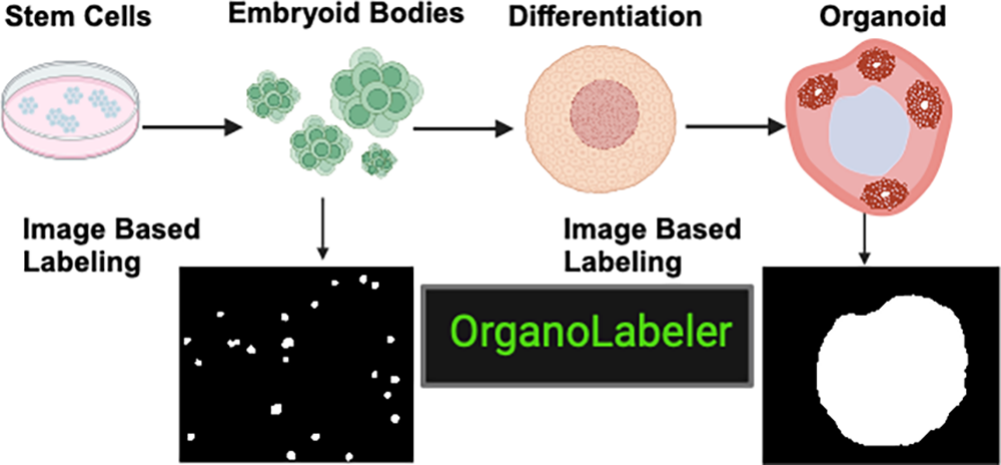

Organoids are self-assembled 3D cellular structures that resemble organs structurally and functionally, providing *in vitro* platforms for molecular and therapeutic studies. Generation of organoids from human cells often requires long and costly procedures with arguably low efficiency. Prediction and selection of cellular aggregates that result in healthy and functional organoids can be achieved by using artificial intelligence-based tools. Transforming images of 3D cellular constructs into digitally processable data sets for training deep learning models requires labeling of morphological boundaries, which often is performed manually. Here, we report an application named OrganoLabeler, which can create large image-based data sets in a consistent, reliable, fast, and user-friendly manner. OrganoLabeler can create segmented versions of images with combinations of contrast adjusting, K-means clustering, CLAHE, binary, and Otsu thresholding methods. We created embryoid body and brain organoid data sets, of which segmented images were manually created by human researchers and compared with OrganoLabeler. Validation is performed by training U-Net models, which are deep learning models specialized in image segmentation. U-Net models, which are trained with images segmented by OrganoLabeler, achieved similar or better segmentation accuracies than the ones trained with manually labeled reference images. OrganoLabeler can replace manual labeling, providing faster and more accurate results for organoid research free of charge.

## 1 Introduction

Organoids are 3D tissue equivalents that provide the structural and functional recapitulation of an organ that can be generated from stromal or pluripotent stem cells.^1^ Organoids provide *in vitro* venues to investigate molecular and mechanistic studies on target organs including realistic models for diseases and drug testing. Advances in stem cell research, bioengineering and organoid technologies lead the generation of diverse portfolio of organoids including the brain, intestine, liver, kidney, stomach, lung, thyroid, and lacrimal gland.^1−12^ Brain organoids (BOs) resemble embryonic development of the cerebellum and can be employed in understanding neurodegenerative diseases and brain research.^13^

Generation of BOs morphologically starts with self-aggregation of cells, followed by formation of embryoid bodies (EB) that hold capacity to differentiate to three distinct germ layers. Neural induction of EBs followed by further differentiation and maturation leads to the formation of BOs. Time to generate the human organoids and costs associated with the process urge the experimental planning and data process to be well optimized minimizing human error.^14^

Machine learning (ML) and deep learning (DL), subfields of artificial intelligence, can be used in every field from health to finance, from education to security, and increase the quality of life.^15^ Traditional ML methods have disadvantages, such as requiring feature extraction. Deep learning methods provide automatic feature extraction and capacity to handle operations demanding high processing power such as image data.^16,17^ In recent years, DL models have been used frequently for segmentation tasks.^18,19^ U-Net^20^ is a DL architecture that has gained significant popularity in medical image segmentation. However, the segmentation performance of these DL models depends on the segmentation quality of the images in the training sets. The performance of these models may be limited due to biases that may occur in images manually tagged by humans.^21^ In biological sciences, images to be used for segmentation tasks can be noisy due to the complexity of the microenvironment of tissues or cells imaged, making labeling by humans difficult or biased.^22^ Moreover, since DL models need to be trained with large data sets to best adapt to real-life scenarios, the process of labeling these data sets is quite time-consuming.^23^ Automatization of the labeling process becomes essential for eliminating human bias and accelerating the process.

There are many image processing and DL-based segmentation studies using biomedical images.^24−28^ Guo et al. combined the Salp Swarm Algorithm, Slime Mold Algorithm, and Differential Evolution algorithms for the analysis of breast cancer pathology images.^24^ Ma et al. used the U-Mamba model, which they developed by combining State Space Sequence Models and Convolutional Neural Networks (CNN), for the segmentation of CT and MR images.^25^ In the study combining Transformers and CNN, Zhang et al. used this tool for polyp, skin lesion, hip, and prostate segmentation tasks.^26^ Wang et al. used Transformers for segmentation of 3D MRI brain tumor images.^27^ Additionally, CellProfiler, which is frequently used in the literature for cell count, size, per-cell protein levels, and morphological analyses, can be used for many biological images from organoids to cells.^28^

A limited number of AI studies have been conducted using organoid images in the literature.^29−34^ Additionally, studies such as OrganoID,^35^ OrgaExtractor,^36^ and OrganoSeg^37^ have been developed to perform morphology analysis using deep learning models (Table 1).

**Table 1:** Image Processing Studies of Organoid Image Segmentation

data set	goal	method	references
brain organoids	segmentation	K-means, SVM	(29)
brain organoids	segmentation	U-Net	(30)
neurons and human mammary gland acinar organoids	clustering and classification	watershed algorithm	(31)
bladder cancer organoids	image processing—texture-based analysis and classification	image features similarity	(32)
bladder cancer organoids	segmentation	U-Net	(33)
mouse-organoid-cells	segmentation	ERF-Net	(34)
colon organoids	segmentation	U-Net	(36)
pancreatic cancer and colon organoids	segmentation	U-Net	(35)
breast cancer spheroid, colon and colorectal-cancer organoid	segmentation—object detection	adaptive and Otsu thresholding	(37)

In this study, we developed OrganoLabeler, a robust, publicly available, and no-code labeling tool based on image processing. OrganoLabeler accelerates the process of generating bias-free ground truth images to be employed in DL models. Its user-adjustable parameters make OrganoLabeler easy to adapt in training set creation tasks for the analysis of organoid and medical images. We have validated increased performance of segmented images created with OrganoLabeler compared to reference manually segmented images.

## 2 Methods

### 2.1 Generation of Brain Organoids from Human-Induced Pluripotent Stem Cells (hiPSC)

Human-induced pluripotent stem cells (hiPSCs) obtained from healthy donors with informed consent are kindly provided from Prof. Tamer Onder.^38^ hiPSC aggregates were plated on 1% Matrigel hESC-Qualified Matrix (Corning, Cat no. 354277)-coated plates and cultured in mTeSR1 (STEMCELL Technologies, Cat no. 85850) with daily medium change. Morphologically differentiated cells were scratched out with a pipet tip. hiPSCs were passaged upon 80% confluency with ReLeSR (STEMCELL Technologies, Cat no. 05872).

For the collection of EBs data sets, embryoid bodies were generated according to protocol based on ref (39). First, hiPSCs were plated as cell clumps on low-attachment plates and cultured with the embryonic stem cell medium (ESCM) composed of DMEM/F-12 (Gibco, Thermo Fisher Scientific, Cat no. 31330-038), 20% KnockOut Serum Replacement (KOSR) (Gibco, Thermo Fisher Scientific, Cat no. 10828028), 1x MEM Non-Essential Amino Acids Solution (MEMNEAA) (Lonza, Cat no. 11140050), 1x GlutaMAX Supplement (Gibco, Thermo Fisher Scientific, Cat no. 35050061), 1% penicillin–streptomycin (Gibco, Thermo Fisher Scientific, Cat no. 15140122), and 0.385 μM 2-mercaptoethanol (Gibco, Thermo Fisher Scientific, 21985023) with the addition of ROCKi (50 μM) (Tocris, Cat no. 1254) for 4–6 days. Bright field images were collected during these time periods.

Brain organoids were generated with a modified version of the protocol of given in ref (39). Briefly, hiPSCs were plated on a low-attachment U-bottom 96 well plate and cultured in ESCM supplemented with ROCKi (50 μM) and a 4 ng/mL Human FGF-basic Recombinant Protein (Gibco, Thermo Fisher Scientific, Cat no. PHG0024). At day 6, formed embryoid bodies (EBs) were transferred into Neural Induction Media (NIM) composed of DMEM/F-12 supplemented with 1x N-2 MAX Media Supplement (R&D Systems, Cat no. AR009), 1x GlutaMAX, 1x MEMNEAA, and 1 μg/mL Heparin (Sigma-Aldrich, Cat no. H3149). At day 10, developed EBs were embedded in a Matrigel Basement Membrane Matrix (Corning, Cat no. 354234) cultured in Cerebral Organoid Differentiation Media (CODM) composed of 1:1 DMEM/F-12 and a Neurobasal Medium (Gibco, Thermo Fisher Scientific, Cat no. 21103049) supplemented with 0.5x N-2 MAX Media Supplement, 1x GlutaMAX, 0.5x MEMNEAA, 1x B-27 Supplement minus vitamin A (Gibco, Thermo Fisher Scientific, Cat no. 12587010), 2.5 μg/mL insulin (Gibco, Thermo Fisher Scientific, Cat no. I9278), and 0.1925 μM 2-mercaptoethanol for 4 days. At the end of the incubation period, 3D clusters were started to incubate in CODM supplemented with B-27 Supplement with vitamin A (Gibco, Thermo Fisher Scientific, Cat no. 17504044). At this step, 3D clusters were differentiated into BOs in three-different culture conditions including a static culture, orbital shaker, and microfluidic chip. Dynamic conditions for the orbital shaker (Miulab GSP-20, P.R.China) were set as 85 rpm mixing and 0.35 μL/min media flow for the microfluidic chip. All groups were cultured for 30 days in a 37 °C incubator under a 5% CO_2_ atmosphere and 95% humidity.

### 2.2 Fabrication of Microfluidic Chips

Microfluidic chips providing dynamic culture conditions for organoids by applying a laminar medium flow were designed with AutoCAD (Autodesk) and CorelDRAW (Corel Co.). Chips were fabricated by stacking poly(methyl methacrylate) (PMMA) layers cut with a laser cutter (Epilog-MINI) and bonded with double side adhesive (3M, 468MP, USA). Sterilization of microfluidic chips was performed by 70% ethanol and 30 min of UV exposure in a laminar flow cabinet.

### 2.3 Immunofluorescence Staining

hiPSC-derived EBs and BOs were fixed with 4% paraformaldehyde (Sigma-Aldrich) for 25 min at room temperature. Brain organoids were embedded in cryomatrix (OCT, Fisher Healthcare) and cryosectioned with Leica CM1950 (Leica Biosystems, Germany). EBs and BOs were permeabilized with permeabilization buffer (0.3% Triton X-100 (neoFroxx, GmbH, Cat no. 8500) (v/v) in PBS (Gibco, Thermo Fisher Scientific, Cat no. P04-36500) for 15 min in room temperature. Then, cells were blocked with blocking buffer (0.05% Tween 20 (Sigma-Aldrich, Cat no. P7949) (v/v), 1% Bovine Serum Albumin (BSA; Sigma-Aldrich, Cat no. A2153) (w/v) in PBS) for 1 h at room temperature. Subsequently, primary antibodies (α-SMA (Cell Signaling Technology, Cat no.48938S), Nestin (Proteintech, Cat no. 19483-1-AP), Sox17 (Abcam, Cat no. Ab84990), Sox2 (Bioss, Cat no. bs-0523R), Tuj1 (R&D Systems, Cat no. MAB1195), and N-cadherin (Cell Signaling Technology, Cat no. 13116T) were added according to related samples and incubated for overnight at 4 °C. Cells were stained with corresponding secondary antibodies for 2 h at room temperature. Nuclei were stained with 4,6-diamidino-2-phenylindole (0.5 μg/mL) (DAPI; Neofroxx, Cat no. 1322). Samples were visualized with a fluorescence microscope (Olympus IX71).

### 2.4 Creation of Data Sets

**Figure 1:**
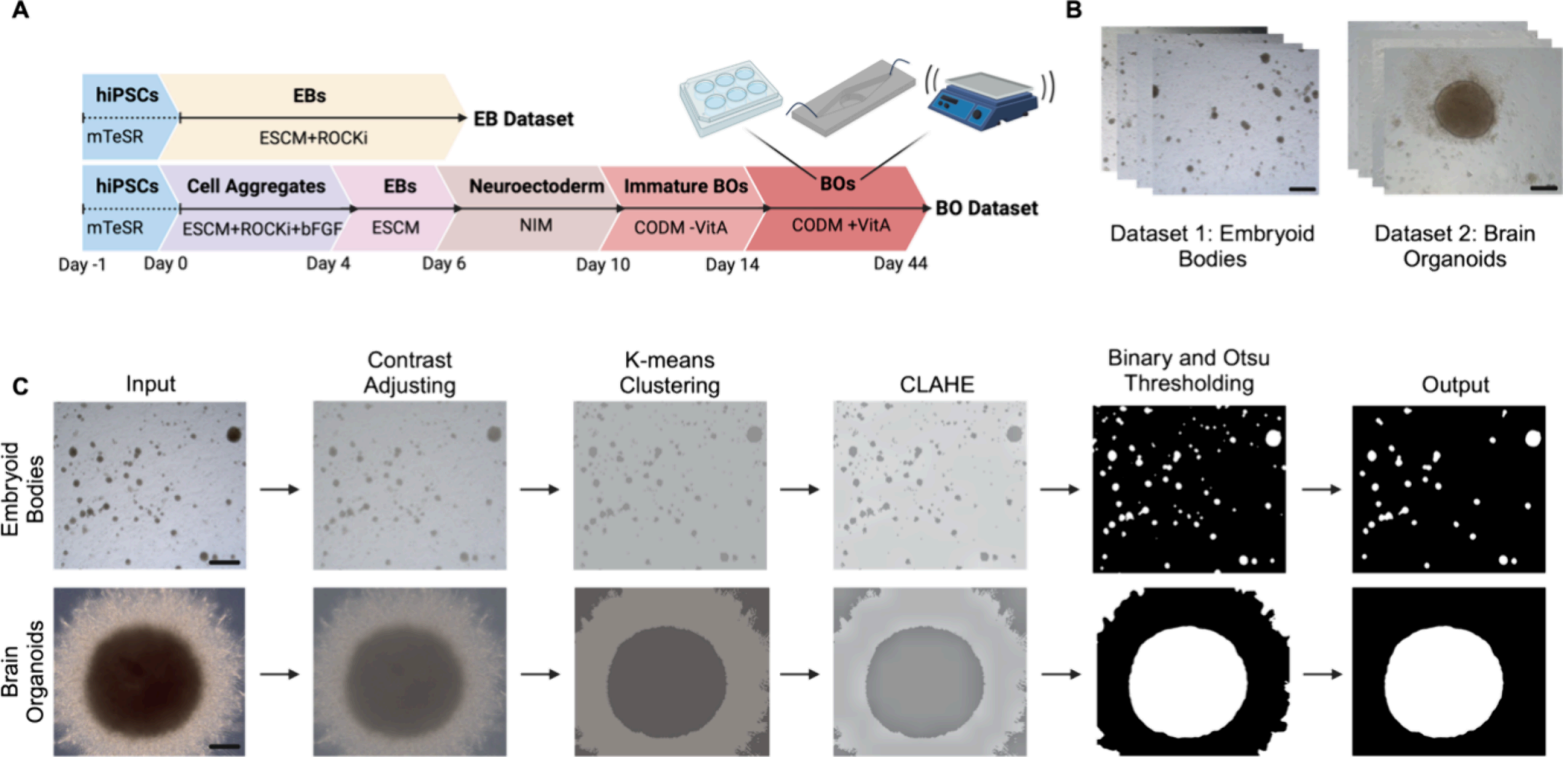
Workflow demonstrating the experimental and computational phases of OrganoLabeler. (A) Schematic illustration of generation of embryoid bodies and brain organoids starting from hiPSCs. (B) Embryoid body and brain organoid data sets created from brightfield microscopy images (scale bars: 200 μm). (C) Image processing steps included in the OrganoLabeler tool.

Experimental protocol outlining the generation of EBs and organoids utilized in creation of data sets used in OrganoLabeler is shown in Figure 1A,B. Brightfield microscopic images with 2464 × 2056 resolution were obtained from day 1 to day 6 for EBs and from day 6 to day 44 for organoids with 10x objective using an inverted microscope equipped with a Zeiss Axiocam 705 color camera (Axiovert Zeiss, Germany). Prior to analyses, images were resized 256 × 256 for convenience. Steps followed for the labeling process of images include contrast adjusting, K-means clustering, CLAHE, binary, and Otsu thresholding methods (Figure 1C). Details of the OrganoLabeler architecture is described in the System Architecture section.

In this study, to demonstrate the generalizability of OrganoLabeler, besides data sets created from our own experimental data, we have included publicly available data sets of intestinal organoids (enteroids), generated by Beck et al.^40^ The EB data set is formed from 165 images, BO data set consists of 133 images, and enteroid data set consists of 299 randomly selected images (Table 2). Segmented images of the brain organoid and embryoid body data set required for comparison with OrganoLabeler outputs were made manually using ImageJ. All data sets are publicly available on Kaggle.

**Table 2:** Datasets Used in the OrganoLabeler System

name	image numbers	microscope
embryoid bodies	165	inverted
brain organoids	133	inverted
enteroids	299	confocal

### 2.5 System Architecture

OrganoLabeler system architecture has been created by merging five different image processing techniques that are contrast adjustment, K-means clustering, contrast-limited adaptive histogram equalization (CLAHE), binary, and Otsu thresholding methods (Figure 1C). First, the input is obtained by the system and contrast adjustment carried out as per entered value. Contrast adjustment plays a vital role for the separation of the background and region(s) of interest (ROI). Then, the OrganoLabeler system performs image segmentation with K-means clustering to be able to separate the foreground object from the background. Because some images have complex backgrounds, the system also performs CLAHE. Before the thresholding step, blurring operation is performed for the better thresholding performance. Finally, ROI(s) are obtained by making binary and Otsu thresholding.

There are five parameters: contrast adjustment, blurring, clip limit, tile grid size for CLAHE, and finally the area limit. Because all these parameters can be adjusted by users, OrganoLabeler gives the best output for users’ expectations.

The whole system is created by the Python programming language (Version 3.9.15). Contrast of the images were adjusted with Python Image Library (Pillow). K-Means clustering, CLAHE, preprocessing, and area size arranging are performed by the OpenCV library. Matplotlibrary is used for visualizations of outputs. Basic mathematical operations are applied by the Numpy library. U-Net models were trained with TensorFlow (2.10.0).

### 2.6 Training Process with U-Net

**Figure 2:**
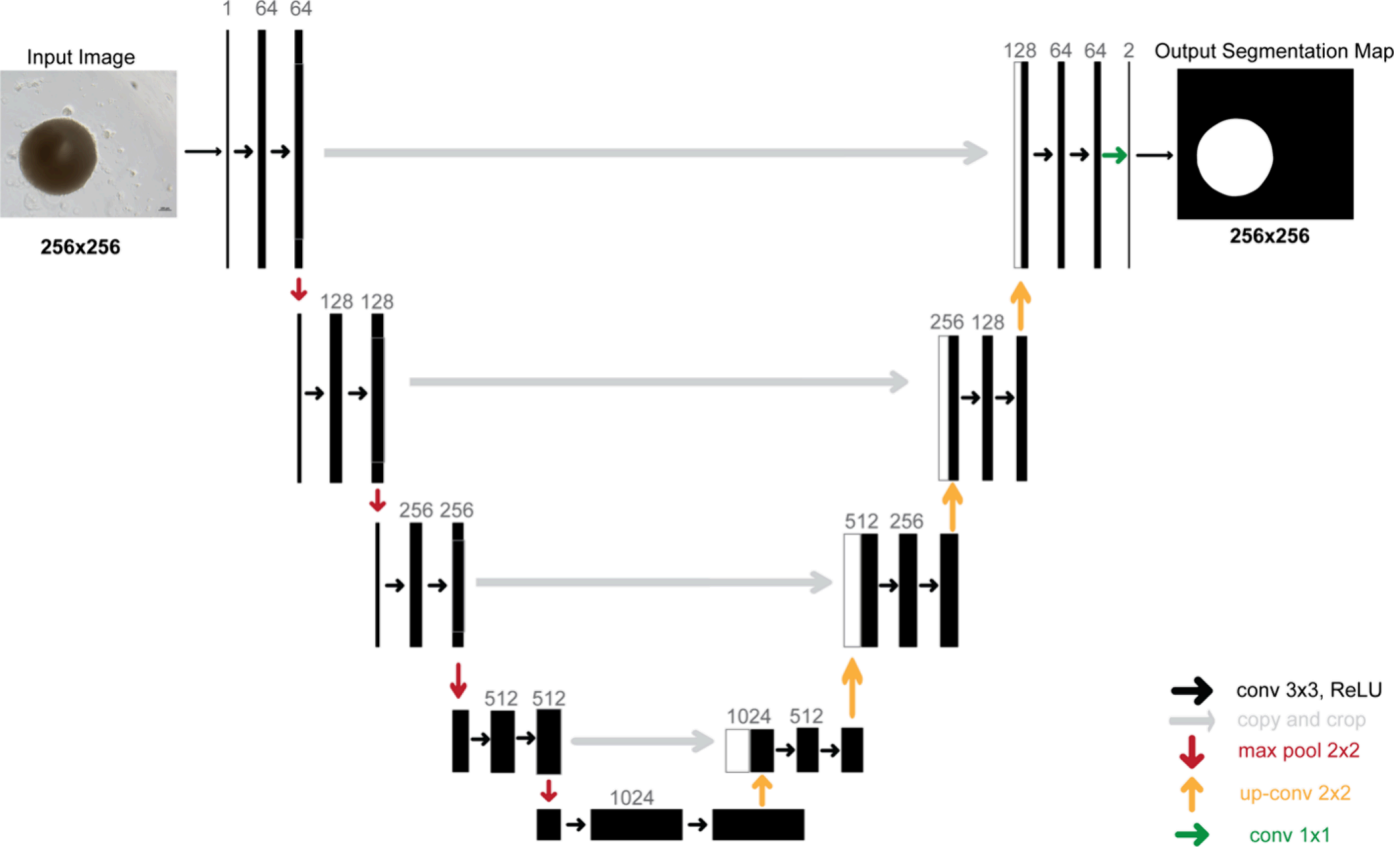
U-Net architecture.

To create the U-Net models,^20^ we used Keras and Tensorflow libraries. Figure 2 shows the U-Net architecture. It comprises repeated double 3 × 3 convolution layers, each followed by a 2 × 2 max pooling operation for down sampling. After each down sampling, the number of feature channels is doubled, which increases up to 1024 at the end of the first half of the architecture. In the second half, each upsampling step is followed by a 2 × 2 up-convolution and a concatenation with the corresponding level of the first half. The final layer uses a convolution to reduce the feature map depth to the desired number of classes.

We resized the data set to 256 × 256 pixel since the U-Net model works at this resolution. In our training, we performed a train and test data set split (80/20%) and we applied 5-fold cross-validation, which corresponds to shifting the test set at each fold.

The loss function is designed as a pixelwise softmax over the final feature map.
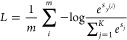
1where *m* is the number of samples (pixels) and *K* is the number of classes. For pixel *i*, *y*^(*i*)^ denotes the correct class label.

We built U-Net models for three-different data sets. The number of images in train and test data set, the number of epochs, and the number of batch sizes are given in Table 3. The experiments in the study were carried out with an Apple MacBook M1Max (64 GB RAM).

**Table 3:** Dataset and Model Information for U-Net

data set name	train–test data set image numbers	epoch	batch size	loss	optimizer	learning rate
embryoid body	131–34	100	8	binary cross-entropy	Adam	1e–3
brain organoid	106–27	20	4	binary cross-entropy	Adam	1e–3
enteroid	239–60	20	4	binary cross-entropy	Adam	1e–3

### 2.7 Evaluation Metrics

#### 2.7.1 Intersection over Union (IoU)

Intersection over Union is an evaluation metric that is used for segmentation and object detection tasks. The IoU metric measures the similarity between two sets of pixels, with one set being the ground truth (true segmentation mask) and the other set being the predicted result (segmentation output of the model). There are three significant terms, namely, true positive (TP), false positive (FP), and false negative (FN), used to calculate IoU for the segmentation task (Table 4).

**Table 4:** Terms for Calculations of IoU and DC

true positive	number of pixels that are correctly classified as object pixels
false positive	number of pixels that are incorrectly classified as object pixels
false negative	number of pixels that are part of the ground truth object but not predicted as being part of an object

The formula of the IoU metric is given below.
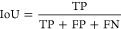


The IoU metric is between 0 and 1, with a value of 1 indicating a perfect segmentation match between the ground truth and predicted result.

#### 2.7.2 Dice Coefficient (DC)

Dice coefficient is a metric that measures the similarity or overlap between two sets. It is used to evaluate the model performance in image segmentation and binary classification. Similar to IoU, its value changes between 0 and 1.

The formula of the DC metric is given below.
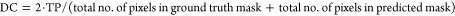


## 3 Results

We developed the OrganoLabeler tool, which quickly segments images of 3D cellular structures, such as embryoid bodies and organoids. Here, we first report the characterization of EBs and BOs and further demonstrate the performance of OrganoLabeler, which has been compared with manually segmented images made by an expert researcher.

### 3.1 Characterization of Embryoid Bodies and Brain Organoids

**Figure 3:**
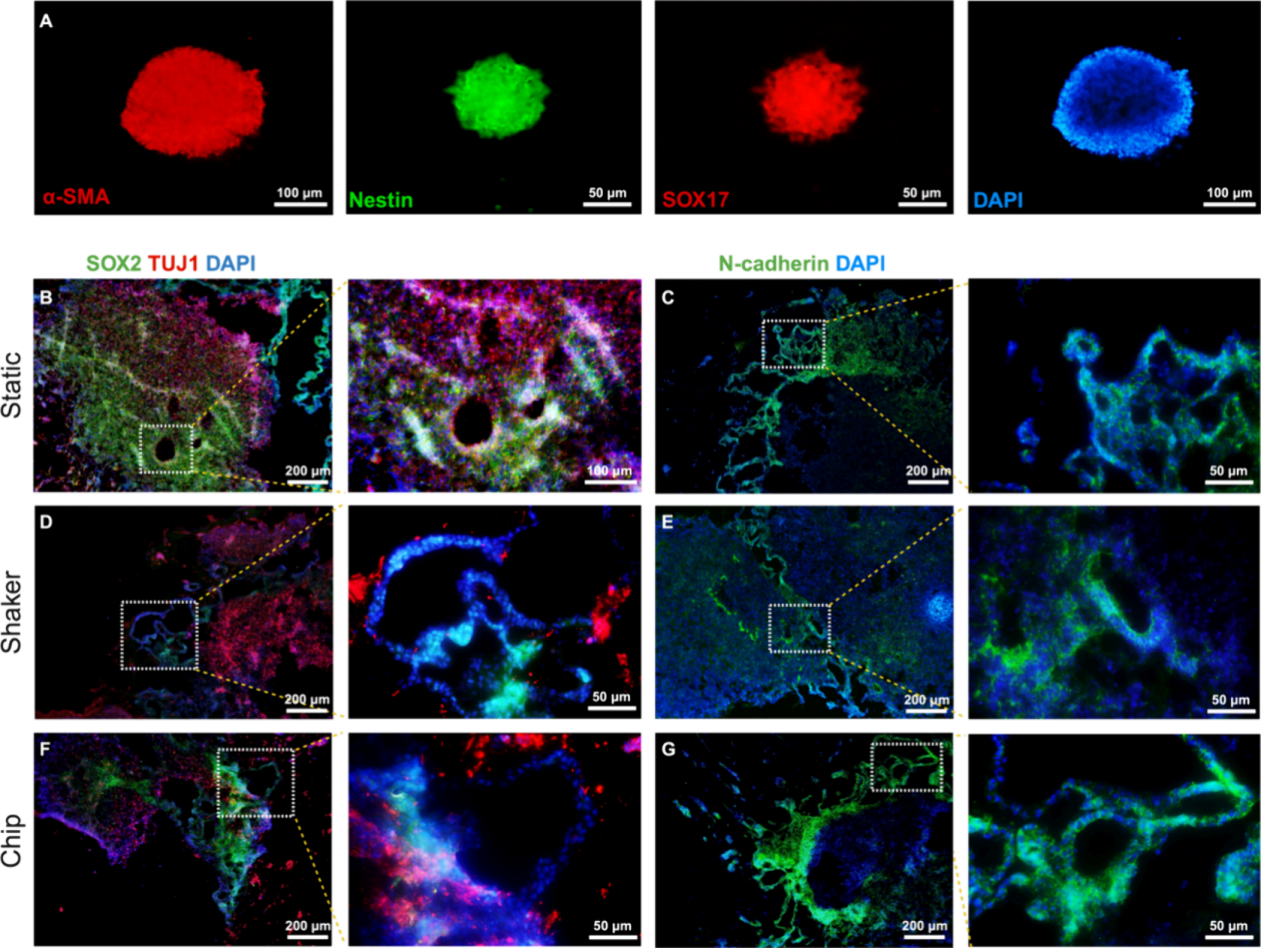
Immunochemical characterization of embryoid bodies and 30 day old brain organoids generated under three-different conditions. (A) Embryoid bodies stained to demonstrate the presence of three distinct germ layers; with α-SMA (mesodermal marker), Nestin (ectodermal marker), Sox17 (endodermal marker), and DAPI (cell nucleus). (B) Brain organoid generated under static condition stained with Sox2 and Tuj1. (C) Brain organoid generated under static condition stained with N-cadherin. (D) Brain organoid generated using an orbital shaker stained with Sox2 and Tuj1. (E) Brain organoid generated using an orbital shaker stained with N-cadherin. (F) Brain organoid generated using a microfluidic chip stained with Sox2 and Tuj1. (G) Brain organoid generated using a microfluidic chip stained with N-cadherin. (Right panels: magnification of dashed zones).

Embryoid bodies are 3D aggregates of stem cells that are capable of generating three germ layers.^41^ EBs were generated from hiPSC in 6 days of suspension culture. We confirm the differentiation capacity of hiPSC into EBs by demonstrating germ layers formed with positivity for α-SMA, Nestin, and Sox17 representing the presence of mesoderm, ectoderm, and endoderm, respectively (Figure 3A). Generated EBs have a diameter with a range of 100–400 μm.

Brain organoids have been shown to be generated under static and dynamic (constant shaking or microfluidic chips) conditions.^39,42,43^ To demonstrate the capacity of OrganoLabeler, here, we generated BOs from hiPSCs either in static culture (Figure 3B,C), or dynamic culture conditions provided by the orbital shaker (Figure 3D,E) or the microfluidic chip (Figure 3F,G). hiPSC-derived BOs were first differentiated toward the ectodermal lineage, which further give rise to a neuronal issue. The neurodifferentiation capacity of generated organoids was achieved through Sox2 expression demonstrating the presence of neural progenitors mostly observed in apical surface of organoids. Tuj1 expressing neurons originated from the neural progenitors localize separately from the progenitor zone. Typical for BOs, N-cadherin is expressed in the apical membrane surrounding cavities, reminiscent of ventricles. Collectively, these data indicate that organoids generated in different conditions have complex heterogeneous regions identifying BOs.

### 3.2 Applying OrganoLabeler to Different Organoid Data Sets

**Figure 4:**
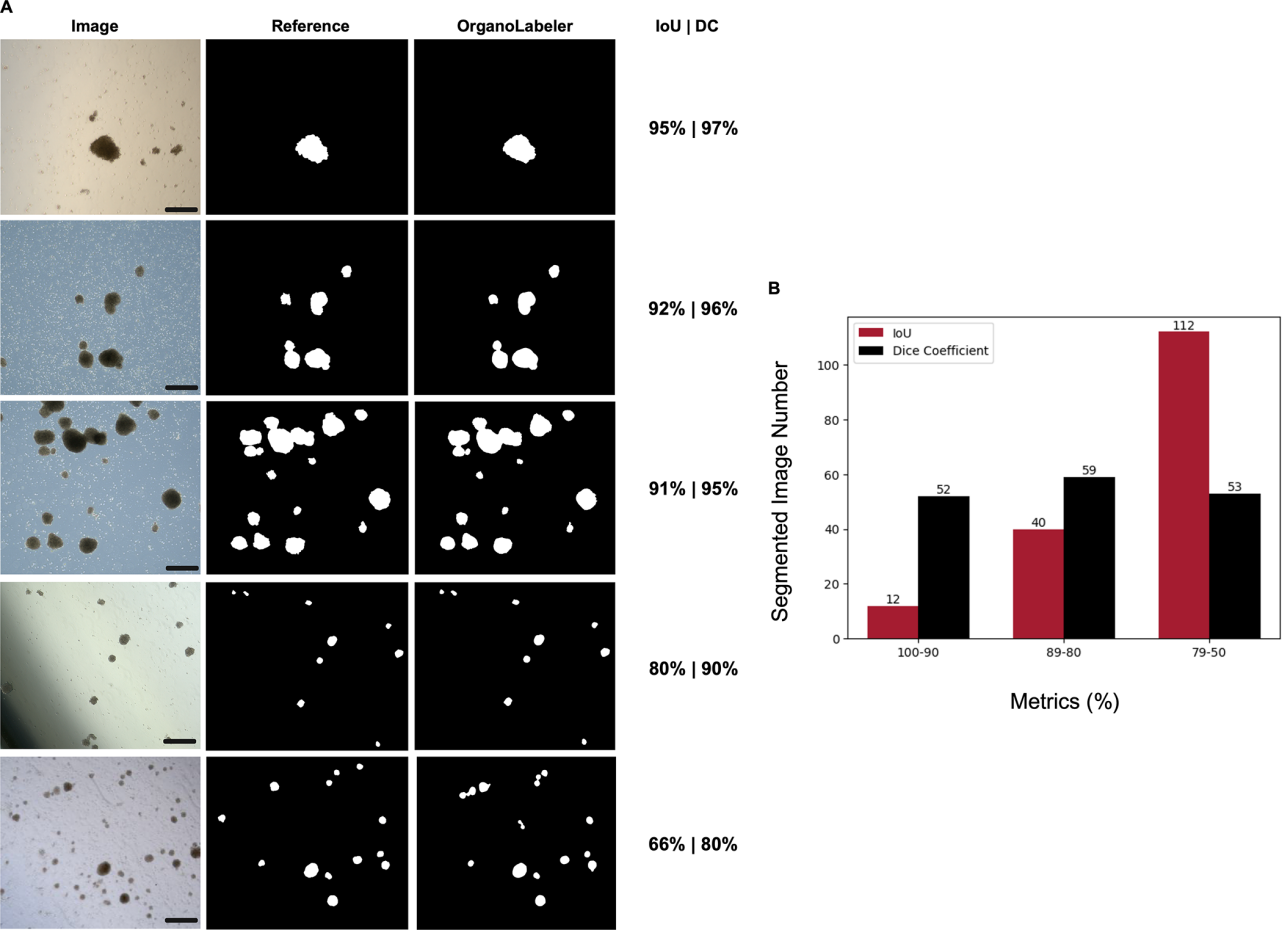
Application of OrganoLabeler to bright field images of embryoid bodies and comparison with reference segmentation. (A) Examples of each evaluation metric distribution from the embryoid data set (scale bars: 200 μm). (B) Metrics ranges of images segmented by OrganoLabeler compared to reference outputs. The *X*-axis shows the ranges of the evaluation metrics, while the *Y*-axis shows the number of segmented images.

Initially, we conducted OrganoLabeler on the data set comprising EBs (Figure 4). We evaluated the outputs obtained with OrganoLabeler using expert-generated reference data and the IoU and DC parameter. Figure 4A shows examples from each evaluation metric range: 100–90, 89–80, and 79–50%. In Figure 4B, the percentage distributions of the outputs obtained from the OrganoLabeler application in the entire data set are provided. Throughout the distribution obtained from the entire data set, the output metrics were concentrated in the 79–50 range in terms of IoU, while they showed a balanced distribution in terms of DC metric. Average IoU and DC were obtained from the EB data set as 71 and 83%, respectively (Table 5).

**Table 5:** Mean IoU and DC Are for OrganoLabeler

data set	data set size	mean IoU (%)	mean dice (%)
embryoid body	165	0.71	0.83
brain organoid	133	0.91	0.92
enteroid	299	0.88	0.94
brain organoid^44^	593	0.86	0.91

**Figure 5:**
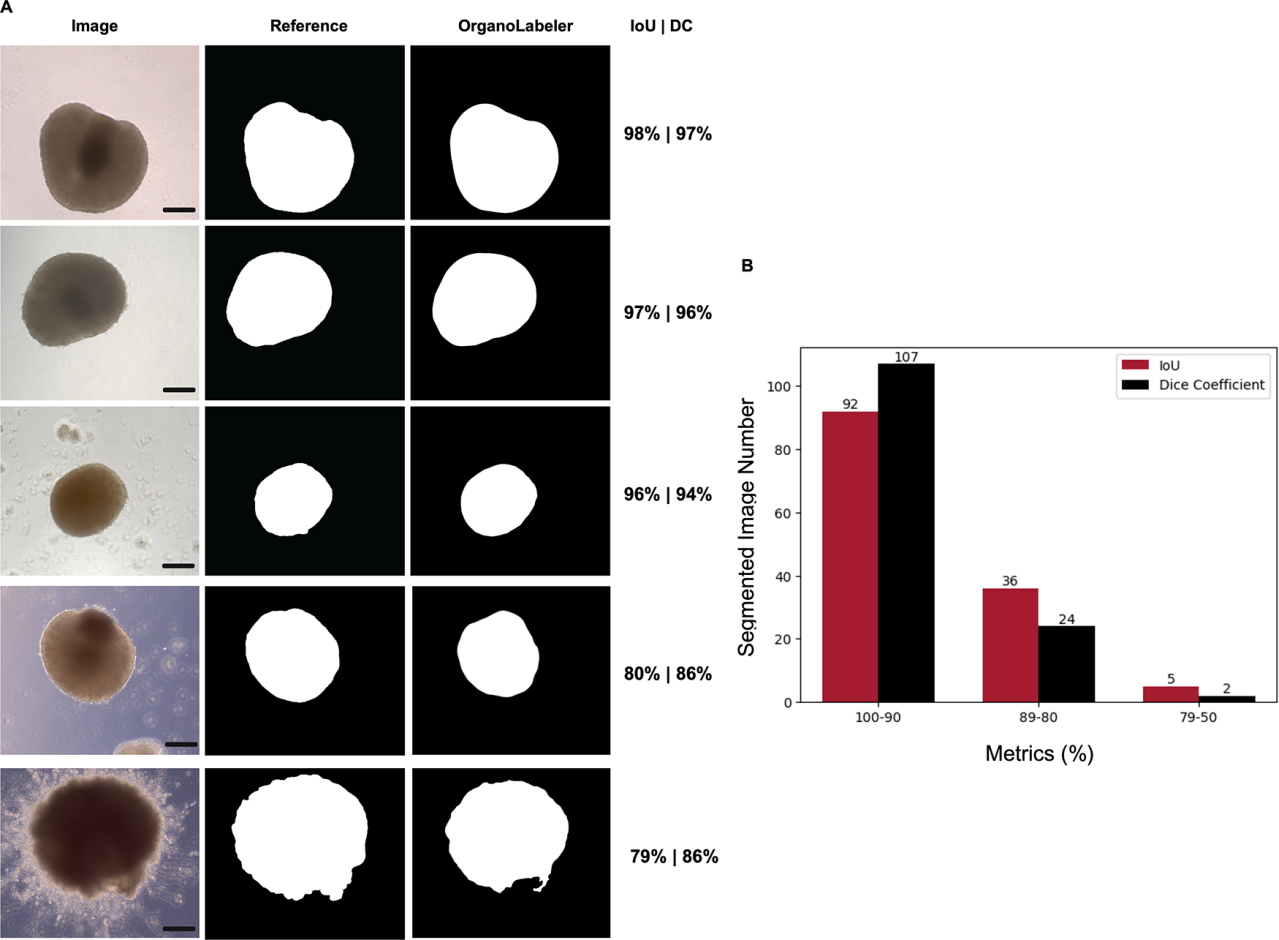
Application of OrganoLabeler to brain organoids and comparison with expert segmentation. (A) Five images with the different IoU and DC in the brain organoid data set (scale bars: 200 μm). (B) IoU and DC ranges of brain organoid images segmented by OrganoLabeler compared to reference outputs. The *X*-axis shows the ranges of the evaluation metrics, while the *Y*-axis shows the number of segmented images.

OrganoLabeler was also applied to the BO data set (Figure 5). The highest IoU and DC obtained from the outputs were determined as 98 and 97%, respectively, and the images with the five different IoU and DC percentages are given in Figure 5A. We obtained the highest number of outputs in the BO data set in the range of 100–90. We found the output numbers in the 50% percentile to be quite low. In addition, the average IoU rate for the entire data set was found to be 91%, while the average DC rate was found to be 92% (Table 5). This indicates that the outputs of OrganoLabeler are like those of the images created by human researchers. More importantly, the difference between the reference images and OrganoLabeler outputs does not affect the performance of deep learning models as will be seen in Section 3.3. This proves OrganoLabeler outputs can be used to train deep segmentation models, which eliminates the need for manually labeled images.

**Figure 6:**
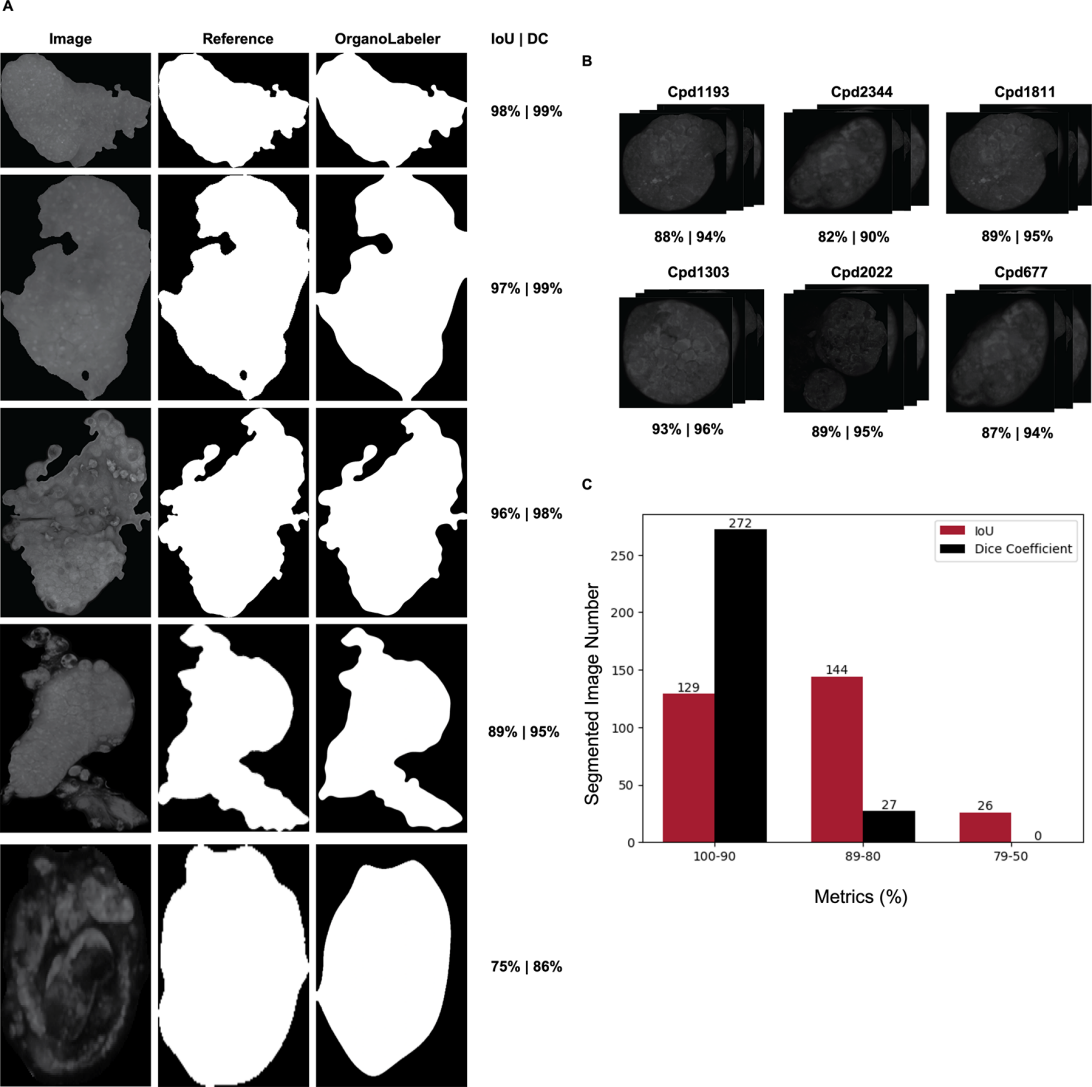
Application of OrganoLabeler to enteroid data set and comparison with reference segmentation. (A) Five images with different distribution percentages of IoU and DC in the enteroid data set. (B) Randomly selected data groups from enteroid data set. (C) IoU and DC ranges of enteroid images segmented by OrganoLabeler compared to reference outputs. The *X*-axis shows the ranges of the evaluation metrics, while the *Y*-axis shows the number of segmented images.

To demonstrate the generalizability of OrganoLabeler, we used the publicly available entoroid data set^40^ (Figure 6). Here, OrganoLabeler showed high performance, similar to in-house generated data sets. Five examples with different percentage distribution of IoU and DC are shown in Figure 6A. OrganoLabeler reached the highest percentage in this data set with an IoU percentage of 98%. The images in the image groups randomly taken from the public data set and the average IoU and DC percentages obtained for each group are given in Figure 6B. When segmented images obtained with OrganoLabeler compared to images created by human researchers, we achieved an average IoU rate of 88% and DC rate of 94%. As shown in Figure 6C, the output from OrganoLabeler is predominantly in the 100–90 range. The part where the outputs are least intense is in the 79–50 range.

Table 5 shows the data set sizes and OrganoLabeler mean IoU and Dice ratios. We have also tested OrganoLabeler in brain organoids generated with iPSCs (Induced Pluripotent Stem Cells) from healthy controls and three-different patient origins.^44^ The segmentation performance of OrganoLabeler was obtained as 86% mean IoU and 91% mean Dice values, respectively. Table 6 compares the performance obtained by OrganoLabeler in all data sets using CellProfiler^28^ and OrganoSeg^37^ segmentation tools.

**Table 6:** Comparing OrganoLabeler Performance with Other Tools

	OrganoLabeler		CellProfiler		OrganoSeg	
data set	mean IoU (%)	mean dice (%)	mean IoU (%)	mean dice (%)	mean IoU (%)	mean dice (%)
embryoid body	0.71	0.83	0.39	0.49	0.62	0.75
brain organoid	0.91	0.92	0.84	0.90	0.86	0.91
enteroid	0.88	0.94	0.66	0.75	0.08	0.13
brain organoid^44^	0.86	0.91	0.83	0.88	0.73	0.82

### 3.3 Comparison of the U-Net Model Trained Using OrganoLabeler Segmented Images with the U-Net Model Trained Using Manually Segmented Images

**Figure 7:**
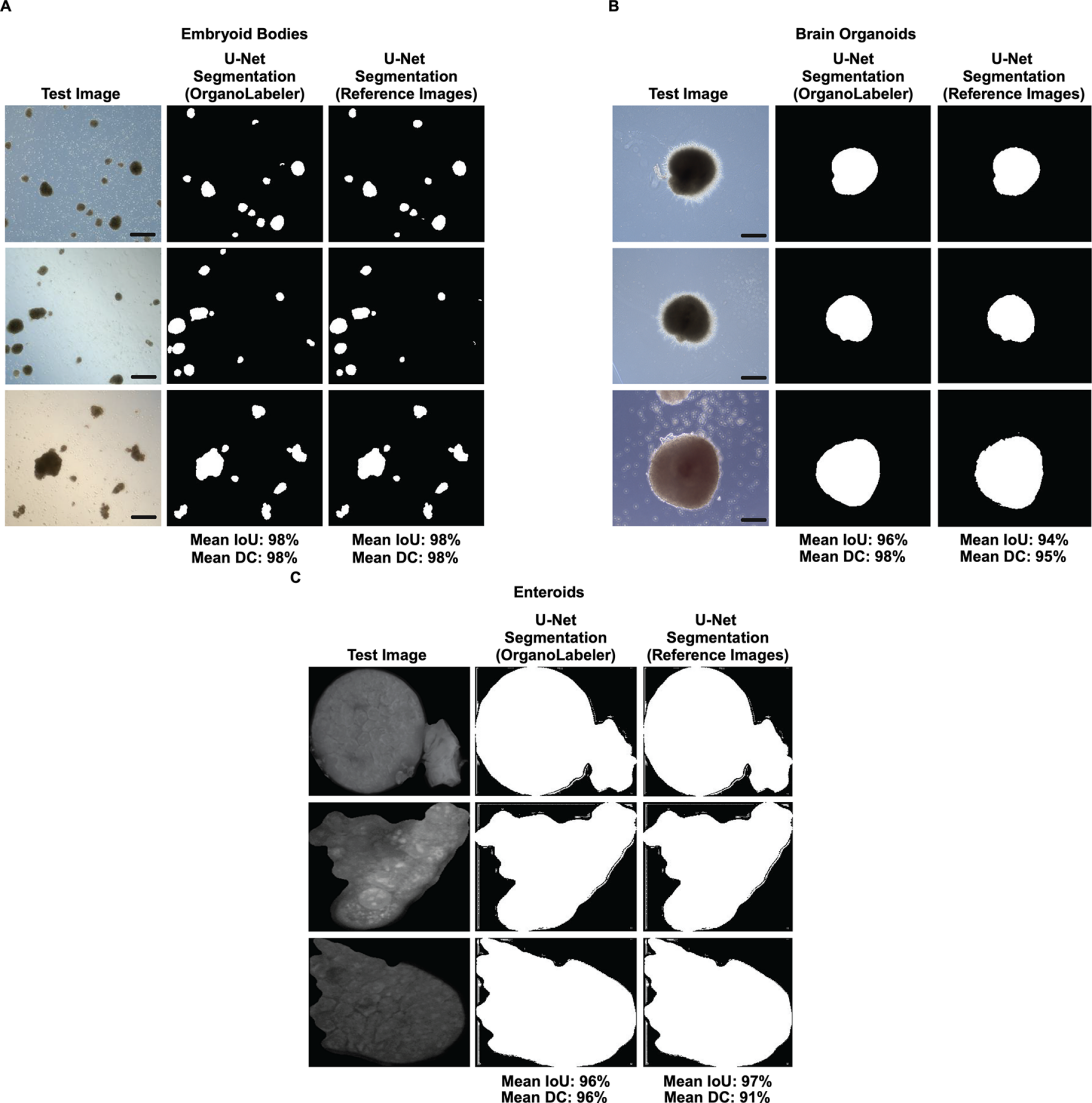
Training the U-Net model with both the data set created by OrganoLabeler and the reference data set and comparing the results. (A) The average IoU and DC values of two different U-Net models trained for embryoid bodies and three randomly selected images and their segmented versions are given (scale bars: 200 μm). (B) Two different U-Net models trained for brain organoids, average IoU and DC values are shown (scale bars: 200 μm). (C) Two different U-Net models trained and tested for the enteroid data set and their average IoU and DC values are given. For the training of each U-Net model, the K-Fold value was determined as 5. Comparative results of three randomly selected images have shown.

For three-different data sets, we trained and tested U-Net models using OrganoLabeler segmented images and manually segmented images (Figure 7). As shown in Figure 7A, two separate U-Net models were trained and tested on the EB data set. One model is trained with images segmented by OrganoLabeler, and the other model is trained with manually segmented (reference) images. Both U-Net models achieved a 98% IoU and DC rates, indicating that OrganoLabeler can replace the manual labeling process of the EB data set. Likewise, two separate U-Net models were trained for the BO data set. Figure 7B shows three randomly selected test images and their segmentation made by (i) U-Net model trained with OrganoLabeler images and (ii) U-Net trained with manually labeled images. The U-Net model trained using images segmented with OrganoLabeler achieved an IoU rate of 96% and a DC rate of 98%, while the U-Net model trained using manually labeled images achieved a 94% IoU rate and 95% DC rate. Lastly, Figure 7C shows the comparison for enteroid images, where U-Net models trained with OrganoLabeler segmented and manually segmented images obtained IoU rates of 96 and 97%, respectively (Table 7). Additionally, the DC rate of OrganoLabeler segmented images is 96%, while the DC rate of manually segmented images is 91%. Results indicate that U-Nets trained with OrganoLabeler output images perform at least as well as those trained with reference data sets. OrganoLabeler can be used to create data sets for training deep learning models (i.e., no need for manual pixel labeling).

**Table 7:** Comparison of U-Net Results of OrganoLabeler Segmented and Reference Images

data sets	U-Net segmentation with OrganoLabeler (IoU %)	U-Net segmentation with reference (IoU %)	U-Net segmentation with OrganoLabeler (DC %)	U-Net segmentation with reference (DC %)
brain organoid	0.96 ± 0.02	0.94 ± 0.03	0.98 ± 0.004	0.95 ± 0.01
embryoid body	0.98 ± 0.004	0.98 ± 0.004	0.98 ± 0.08	0.98 ± 0.008
enteroid	0.96 ± 0.01	0.97 ± 0.01	0.96 ± 0.01	0.91 ± 0.08

## 4 Discussion

Organoids are 3D tissues that recapitulate structural and functional features of organs and can be widely utilized in life sciences and health applications such as disease modeling, drug screening, personalized medicine, and developmental biology.^1^ Similar to other tissue organoids, brain organoids derived from hiPSCs have 3D morphologies formed in complex cellular composition. Starting from cell aggregates, the differentiation process toward brain organoids takes weeks, even months, with costly laborious tasks. Although applying identical differentiation protocols, the yield of generation brain organoids with distinct histological characterization may differ, indicating not all cell aggregates succeed toward the targeted organoid. Utilization of image-based analytical tools may assist in selecting morphologically potent 3D cell clusters at the early phase, eliminating the number of samples and thereby minimizing expenses.

Here, we report an automated image labeling tool named as OrganoLabeler, which can be utilized further in segmentation of data sets used in artificial intelligence-based predictive models. Such labeling tools are essential to digitalize wet lab-generated biological results into a processable data set in image-based computational analyses. In this study, we first generated embryoid bodies that are precursors of organoids and further obtained brain organoids as relevant 3D biological samples to be employed in labeling of image data sets.

Embryoid bodies are 3D cellular structures formed during the generation of brain organoids. Their cellular composition initiates the further differentiation toward a specific type of tissue, considering the germ layer origin. We have accordingly characterized the hiPSCs-derived EBs for mesoderm, ectoderm, and endoderm with positivity to α-SMA, Nestin, and SOX17, respectively. Brain organoids were generated under static and dynamic settings using three-different culture conditions as previously reported.^39,43,45,46^ We demonstrate the successful differentiation of hiPSCs into BO where organoids from all conditions show positivity for neuroprogenitor SOX2 and neuronal marker TUJ1 through immunohistochemical characterization (Figure 3). Zonal cavities representing the ventricle structures present in BOs have been identified with N-Cadherin staining. Independent from culture conditions, we obtained BOs to be used as model organoids for labeling.

Although there are several labeling tools for deep learning models, these tools are both time-consuming and prone to human-based errors because they require manual labeling.^47−49^ However, OrganoLabeler differs from these as a system that automates the image labeling process. Morphological tracking of organoids is of great importance in the process of their creation. Image processing and deep-learning-based approaches can be used for this tracking. Studies using image processing or DL models for the analysis of images obtained from organoids have been increasing in recent years.^29−34^ Mergenthaler et al. used the maximum correlation thresholding and watershed algorithm methods to determine contours in organoid images.^31^ OrganoLabeler combined the more standard thresholding method and three-different methods. Camalan et al. used image filtering for image texture analysis and created cumulative histograms to identify the most correlated images.^32^ They created a cancer grading system that they trained with two different data sets. OrganoLabeler combines five different image processing methods to obtain segmented images with user-defined parameters.

Additionally, tools for morphological analysis of organoids have been developed using deep learning models.^35,36^ Convolutional neural networks were used for the segmentation of organoids in the tool developed by Matthews et al. They trained their system with a pancreatic organoid data set.^35^ Segmentation was carried out with the U-Net model in OrgaExtractor developed by Park et al.^36^ Colon organoid was used as data set in the study. However, training of these models is laborious and challenging due to the fact that the segmented images have to be prepared manually. In our study, we developed a no-code automated tool called OrganoLabeler.

OrganoLabeler was tested on a brain organoid data set generated from one healthy control iPSC and three patient-derived iPSCs.^44^ As a result of the analysis, segmented images obtained from OrganoLabeler were compared with reference segmented images, and 86% mean IoU and 91% mean Dice values were obtained. Testing OrganoLabeler on four different brain organoids, three of which were generated from patient-derived iPSCs, as well as our own brain organoids, contributes to its generalizability.

The performance of OrganoLabeler was compared with two different tools called CellProfiler^28^ and OrganoSeg.^37^ These tools include different image processing techniques and perform image processing-based analyses. CellProfiler performs image processing analyses using a combination of many different methods. However, since this tool contains a large number of methods, it is difficult to find the appropriate combination of them. In addition, finding the appropriate parameters of the methods can be quite time-consuming. OrganoSeg, on the other hand, was quite insufficient in some data sets with its limited number of adjustable parameters (three parameters) (Table 6). OrganoLabeler, on the other hand, consists of a combination of five different image processing techniques. Finding the appropriate values with five different parameters is relatively less time-consuming.

In OrganoSeg, after segmentation was performed with default parameters, the parameters were changed specifically for the data set for better segmentation. The results obtained were compared with the reference data sets (Table 6). CellProfiler gave the closest performance to OrganoLabeler in brain organoids. In embryoid bodies and enteroids, the OrganoLabeler performance was better. In OrganoSeg, performance closer to that of OrganoLabeler was obtained in embryoid bodies compared to CellProfiler. However, in brain organoids, the performance came close to that of OrganoLabeler as a result of the tuning process of the default values. Unlike other tools, quite a bad performance was obtained in enteroids despite parameter tuning. This may be due to the different image types of enteroids and some images being close to the background. In addition, the possibility of making thresholding inverted in OrganoLabeler and CellProfiler has a quite positive effect on the enteroid segmentation performance.

To test our tool, we used three-different data sets and trained U-Net models using the resulting segmented images. We also trained U-Net models using manually labeled images and compared the results. The U-Net models trained with segmented images generated with OrganoLabeler mostly outperformed the models trained with manually segmented images.

Two of the data sets used included mature BOs from EBs summarizing the organoid development process, while the other was a data set generated with enteroid images. The embryoid body data set contains 165 images, the BO data set contains 133 images, and the enteroid data set contains 299 images. Thus, OrganoLabeler was tested with different organoid types and mostly exceeded the success of manually labeled data. Moreover, it accelerated the labeling process considerably.

OrganoLabeler has a few limitations. In the segmentation of small EBs in the EB data set, one of the three data sets used, our system had difficulty in segmenting appropriately. In addition, there were some images taken with inverted microscopes of the EBs in the images taken from the boundary parts of the EBs where the EBs were located, which tended to be incorrectly segmented due to the shadow created by the wells. In addition, in a small number of images containing this shadow, the EBs coming into the shadow part could not be segmented by the system.

## 5 Conclusions

In this study, we developed OrganoLabeler, a tool that can generate segmented organoid images to be used for DL models of cell and cellular structures. We compared the performance of OrganoLabeler with images manually segmented by experts. OrganoLabeler outputs are similar enough to the manually segmented images so that they can be used to train deep segmentation convolutional neural networks. We proved that by training a state-of-the-art segmentation CNN (U-Net). U-Net models trained with segmented images generated with OrganoLabeler mostly outperformed the models trained with manually segmented images. Being user-friendly, OrganoLabeler is available free of charge with user-adjustable parameters. This makes it easy to create fast and human error-free data sets for segmentation models to be trained with organoid images.

## Data Availability

All codes and datasets are available in Kaggle. https://www.kaggle.com/datasets/burakkahveci/brain-organoid-and-embryoid-bodies-organolabeling

## References

[ref1] Zhao Z.; et al. Organoids. Nat. Rev. Methods Primers 2022, 2 (1), 94 10.1038/s43586-022-00186-8. 37325195 PMC10270325

[ref2] Kim J.; Koo B. K.; Knoblich J. A. Human organoids: model systems for human biology and medicine. Nat. Rev. Mol. Cell Biol. 2020, 21 (10), 571–584. 10.1038/s41580-020-0259-3. 32636524 PMC7339799

[ref3] Hayashi R.; et al. Generation of 3D lacrimal gland organoids from human pluripotent stem cells. Nature 2022, 605 (7908), 126–131. 10.1038/s41586-022-04613-4. 35444274

[ref4] Asal M.; et al. Development of lacrimal gland organoids from iPSC derived multizonal ocular cells. Front. Cell Dev. Biol. 2023, 10, 1058846 10.3389/fcell.2022.1058846. 36684423 PMC9846036

[ref5] Schwank G.; et al. Functional repair of CFTR by CRISPR/Cas9 in intestinal stem cell organoids of cystic fibrosis patients. Cell Stem Cell 2013, 13 (6), 653–8. 10.1016/j.stem.2013.11.002. 24315439

[ref6] Matano M.; et al. Modeling colorectal cancer using CRISPR-Cas9–mediated engineering of human intestinal organoids. Nature Medicine 2015, 21 (3), 256–262. 10.1038/nm.3802. 25706875

[ref7] Takasato M.; et al. Kidney organoids from human iPS cells contain multiple lineages and model human nephrogenesis. Nature 2015, 526 (7574), 564–568. 10.1038/nature15695. 26444236

[ref8] Broutier L.; et al. Culture and establishment of self-renewing human and mouse adult liver and pancreas 3D organoids and their genetic manipulation. Nat. Protoc. 2016, 11 (9), 1724–1743. 10.1038/nprot.2016.097. 27560176

[ref9] Bartfeld S.; Clevers H. Organoids as Model for Infectious Diseases: Culture of Human and Murine Stomach Organoids and Microinjection of Helicobacter Pylori. J. Visualized Exp. 2015, 105, e53359 10.3791/53359-v. PMC4692704 26650279

[ref10] Dye B. R.; et al. In vitro generation of human pluripotent stem cell derived lung organoids. eLife 2015, 4, e05098 10.7554/eLife.05098. 25803487 PMC4370217

[ref11] Ogundipe V. M. L.; et al. Generation and Differentiation of Adult Tissue-Derived Human Thyroid Organoids. Stem Cell Reports 2021, 16 (4), 913–925. 10.1016/j.stemcr.2021.02.011. 33711265 PMC8072035

[ref12] Koçak G.; et al. Generation of Anterior Segment of the Eye Cells from hiPSCs in Microfluidic Platforms. Adv. Biol. 2024, 8 (5), 2400018 10.1002/adbi.202400018. 38640945

[ref13] Lancaster M. A.; et al. Cerebral organoids model human brain development and microcephaly. Nature 2013, 501 (7467), 373–379. 10.1038/nature12517. 23995685 PMC3817409

[ref14] Bose S.; Clevers H.; Shen X. Promises and Challenges of Organoid-Guided Precision Medicine. Med. (N Y) 2021, 2 (9), 1011–1026. 10.1016/j.medj.2021.08.005. PMC8492003 34617071

[ref15] Triantafyllidis A. K.; Tsanas A. Applications of Machine Learning in Real-Life Digital Health Interventions: Review of the Literature. J. Med. Internet Res. 2019, 21 (4), e12286 10.2196/12286. 30950797 PMC6473205

[ref16] LeCun Y.; Bengio Y.; Hinton G. Deep learning. Nature 2015, 521 (7553), 436–444. 10.1038/nature14539. 26017442

[ref17] Tekir S.; Yalin B. Deep Learning: Exemplar Studies in Natural Language Processing and Computer Vision. In Data Mining: Methods, Applications and Systems, Derya B., Ed. IntechOpen: Rijeka, 2020, p. 1.

[ref18] Minaee S.; et al. Image Segmentation Using Deep Learning: A Survey. IEEE Trans. Pattern Anal. Mach. Intell. 2022, 44 (7), 3523–3542. 10.1109/TPAMI.2021.3059968. 33596172

[ref19] Rizwan I Haque I.; Neubert J. Deep learning approaches to biomedical image segmentation. Informatics in Medicine Unlocked 2020, 18, 100297 10.1016/j.imu.2020.100297.

[ref20] Ronneberger O.; Fischer P.; Brox T. U-net: Convolutional networks for biomedical image segmentation. In Medical Image Computing and Computer-Assisted Intervention – MICCAI 2015: 18th International Conference, Munich, Germany, October 5-9, 2015, Proceedings, Part III; Springer, 2015.

[ref21] Zhang L.; et al. Disentangling human error from ground truth in segmentation of medical images. Adv. Neural Inf. Process. Syst. 2020, 33, 15750–15762.

[ref22] Katkar J.; Baraskar T.; Mankar V. R. A novel approach for medical image segmentation using PCA and K-means clustering. In 2015 International Conference on Applied and Theoretical Computing and Communication Technology (iCATccT); IEEE, 2015.

[ref23] Ratner A. J.; et al. Data programming: Creating large training sets, quickly. Adv. Neural Inf. Process. Syst. 2016, 29, 3567–3575. 29872252 PMC5985238

[ref24] Guo H.; et al. Multi-threshold Image Segmentation based on an improved Salp Swarm Algorithm: Case study of breast cancer pathology images. Computers in Biology and Medicine 2024, 168, 107769 10.1016/j.compbiomed.2023.107769. 38039898

[ref25] Ma J.; Li F.; Wang B. U-mamba: Enhancing long-range dependency for biomedical image segmentation arXiv, 2024. DOI:10.48550/arXiv.2401.04722

[ref26] Zhang Y.; Liu H.; Hu Q. TransFuse: Fusing Transformers and CNNs for Medical Image Segmentation; Springer International Publishing: Cham, 2021.

[ref27] Wang W. TransBTS: Multimodal Brain Tumor Segmentation Using Transformer; Springer International Publishing: Cham, 2021.

[ref28] Carpenter A. E.; et al. CellProfiler: image analysis software for identifying and quantifying cell phenotypes. Genome Biol. 2006, 7 (10), R100 10.1186/gb-2006-7-10-r100. 17076895 PMC1794559

[ref29] Brémond-Martin C. TDA-Clustering Strategies for the Characterization of Brain Organoids; Springer Nature Switzerland: Cham, 2022.

[ref30] Brémond Martin C.; et al. Mu-Net a Light Architecture for Small Dataset Segmentation of Brain Organoid Bright-Field Images. Biomedicines 2023, 11 (10), 2687 10.3390/biomedicines11102687. 37893062 PMC10603975

[ref31] Mergenthaler P.; et al. Rapid 3D phenotypic analysis of neurons and organoids using data-driven cell segmentation-free machine learning. PLoS Comput. Biol. 2021, 17 (2), e1008630 10.1371/journal.pcbi.1008630. 33617523 PMC7932518

[ref32] Camalan S. Bladder cancer organoid image analysis: textured-based grading. In Medical Imaging 2021: Digital Pathology; SPIE, 2021.

[ref33] Zhang S.; et al. A deep learning model for drug screening and evaluation in bladder cancer organoids. Front. Oncol. 2023, 13, 1064548 10.3389/fonc.2023.1064548. 37168370 PMC10164950

[ref34] Lalit M.; Tomancak P.; Jug F. EmbedSeg: Embedding-based Instance Segmentation for Biomedical Microscopy Data. Medical Image Analysis 2022, 81, 102523 10.1016/j.media.2022.102523. 35926335

[ref35] Matthews J. M.; et al. OrganoID: A versatile deep learning platform for tracking and analysis of single-organoid dynamics. PLoS Comput. Biol. 2022, 18 (11), e1010584 10.1371/journal.pcbi.1010584. 36350878 PMC9645660

[ref36] Park T.; et al. Development of a deep learning based image processing tool for enhanced organoid analysis. Sci. Rep. 2023, 13 (1), 19841 10.1038/s41598-023-46485-2. 37963925 PMC10646080

[ref37] Borten M. A.; et al. Automated brightfield morphometry of 3D organoid populations by OrganoSeg. Sci. Rep. 2018, 8 (1), 5319 10.1038/s41598-017-18815-8. 29593296 PMC5871765

[ref38] Akbari S.; et al. Robust, Long-Term Culture of Endoderm-Derived Hepatic Organoids for Disease Modeling. Stem Cell Reports 2019, 13 (4), 627–641. 10.1016/j.stemcr.2019.08.007. 31522975 PMC6829764

[ref39] Lancaster M. A.; Knoblich J. A. Generation of cerebral organoids from human pluripotent stem cells. Nat. Protoc. 2014, 9 (10), 2329–2340. 10.1038/nprot.2014.158. 25188634 PMC4160653

[ref40] Beck L. E.; et al. Systematically quantifying morphological features reveals constraints on organoid phenotypes. Cell Syst. 2022, 13 (7), 547–560.e3. 10.1016/j.cels.2022.05.008. 35705097 PMC9350855

[ref41] Jaenisch R.; Young R. Stem cells, the molecular circuitry of pluripotency and nuclear reprogramming. Cell 2008, 132 (4), 567–82. 10.1016/j.cell.2008.01.015. 18295576 PMC4142810

[ref42] Wang Y.; et al. Engineering stem cell-derived 3D brain organoids in a perfusable organ-on-a-chip system. RSC Adv. 2018, 8 (3), 1677–1685. 10.1039/C7RA11714K. 35540867 PMC9077091

[ref43] Saglam-Metiner P.; et al. Spatio-temporal dynamics enhance cellular diversity, neuronal function and further maturation of human cerebral organoids. Commun. Biol. 2023, 6 (1), 173 10.1038/s42003-023-04547-1. 36788328 PMC9926461

[ref44] Schröter J.; et al. A large and diverse brain organoid dataset of 1,400 cross-laboratory images of 64 trackable brain organoids. Sci. Data 2024, 11 (1), 514 10.1038/s41597-024-03330-z. 38769371 PMC11106320

[ref45] Goto-Silva L.; et al. Computational fluid dynamic analysis of physical forces playing a role in brain organoid cultures in two different multiplex platforms. BMC Dev. Biol. 2019, 19 (1), 3 10.1186/s12861-019-0183-y. 30841924 PMC6404276

[ref46] Kelava I.; et al. Androgens increase excitatory neurogenic potential in human brain organoids. Nature 2022, 602 (7895), 112–116. 10.1038/s41586-021-04330-4. 35046577 PMC7612328

[ref47] Russell B. C.; et al. LabelMe: A Database and Web-Based Tool for Image Annotation. International Journal of Computer Vision 2008, 77 (1), 157–173. 10.1007/s11263-007-0090-8.

[ref48] Tkachenko M.; Malyuk M.; Holmanyuk A; Liubimov N Label Studio: Data labeling software, 2020-2022.

[ref49] Tancre P. LabelBox*:* https://labelbox.com/. 2018.

